# Seasonal Variability of Lipophilic Compounds in Oat (*Avena sativa* L.) Straw: A Comprehensive Chemical Study

**DOI:** 10.1021/acs.jafc.4c05002

**Published:** 2024-09-03

**Authors:** Gisela Marques, Ana Gutiérrez, Francisco Barro, José C. del Río, Jorge Rencoret

**Affiliations:** † Instituto de Recursos Naturales y Agrobiología de Sevilla (IRNAS-CSIC), Avenida Reina Mercedes 10, E-41012 Seville, Spain; ‡ Instituto de Agricultura Sostenible (IAS-CSIC), Avenida Menéndez Pidal s/n, E-14004 Córdoba, Spain

**Keywords:** oat straw, high-molecular-weight esters, fatty acids, steroids, waste valorization, seasonal variation

## Abstract

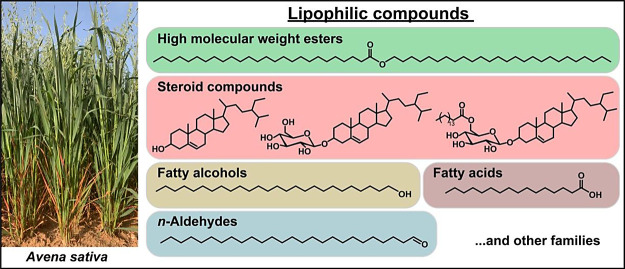

Oat straw, a residue of *Avena sativa* L., is recognized for its abundance in cellulose, hemicelluloses, and lignin. However, its potential as a source of lipophilic compounds within the framework of a biorefinery concept still remains unexplored. In this study, we conducted an extensive investigation into the content and chemical composition of the lipophilic compounds present in acetone extracts from oat straws of two distinct oat varieties, namely, Karen and Isaura. Furthermore, we examined their seasonal variability in content and composition in straw samples from oats planted in both spring and winter seasons. The extracted lipophilic compounds were predominantly composed of high molecular weight esters (26.0–38.1%), steroids (16.6–24.0%), *n*-fatty alcohols (10.9–20.7%), *n*-fatty acids (10.9–16.0%), and *n*-aldehydes (10.7–15.8%), with lower amounts of *n*-alkanes (1.1–3.0%), acylglycerides (2.3–3.8%), phytol and phytyl esters (0.6–2.9%), β-diketones (0.1–2.5%), triterpenoids (0.9–1.2%), tocopherols and tocopheryl esters (0.2–0.7%), 2-hydroxy fatty acids (0.1–0.2%), and *n*-alkylresorcinols (0.1%). Notably, these different classes of compounds exhibited variations in their contents depending on the oat variety and the specific planting season. Of particular interest was the Karen variety, which presented significant amounts of high molecular weight esters, free fatty acids, and acylglycerols, especially when it was cultivated during the winter season. These findings underline the potential of oat straw as a valuable resource for lipid extraction within a biorefinery context and emphasize the importance of selecting the appropriate variety and season for optimal lipid yield.

## Introduction

Oat (*Avena sativa* L.) is a cereal grain that holds a prominent place in the agricultural landscape, renowned for its nutritional value, versatility in culinary applications, and numerous health benefits.^1^ Beyond its importance as a food source, oats have garnered attention in recent years for their potential in the sustainable production of biobased materials and bioenergy, mainly due in large part to the biomass-rich residue that remains after grain harvest—oat straw. Oat straw is the aerial component of the oat plant and remains after the grains are harvested. Although often considered an agricultural waste, oat straw possesses intrinsic value and holds considerable promise in the context of agro-biorefineries. Oat straw is primarily composed of cellulose, hemicelluloses, and lignin,^2,3^ which offers the potential for conversion into a variety of valuable products, including biofuels, biopolymers, and chemicals.^4−6^ Moreover, oat straw also contains other nonstructural components, such as the extractives, that are easily obtained from biomass, and depending on their composition might have great appeal as “green” chemicals in the pharmaceutical, cosmetic, food, and biological/chemical industries.^7−11^ According to their solubility, extractives can be divided into lipophilic (obtained with nonpolar or low polar solvents) and polar/hydrophilic (obtained with polar solvents). Lipophilic extractives comprise a diverse and heterogeneous group of compounds that include alkanes, fatty alcohols, fatty acids, resin acids, acylglycerides, high molecular weight ester waxes, terpenes, and steroids, among others. Oat straw presents around 2% of lipophilic extractives that can also be valorized.^2^

While the lipid composition of oats has been thoroughly studied across various plant parts, the predominant focus of research has been on the grain,^12−18^ leaving a noticeable gap in research concerning the lipids present in the straw. Despite numerous studies describing the lipid composition of other cereal straws, such as rice and wheat,^19,20^ only one previous work has reported the composition of oat leaf wax that included hydrocarbons, esters, free alcohols, free acids, β-diketones and hydroxy-β-diketones.^21^ For this reason, this study presents a comprehensive study of the lipophilic fractions extracted from the straws of two distinct oat varieties (Karen and Isaura) cultivated in two different seasons (winter and spring). The aim is to explore the effects of seasonal variation and genetic diversity in their composition.

## Materials and Methods

### Oat Straw Samples

Two oat varieties, namely, Karen (obtained from a Previsión×Alcudia crossing) and Isaura (resulting from a Pedigreed No. 7×Alcudia crossing), were selected for this study. Additional details of these varieties are published elsewhere.^2^ Both oat varieties were cultivated in two distinct seasons, winter and spring, in an experimental field located in Córdoba (South Spain), during the agricultural year 2020–2021. Upon reaching maturity, the oat plants were harvested and their straws were collected. Subsequently, the straw samples were subjected to air-drying at room temperature, until a constant weight was achieved. The dried straw samples were finely ground to pass through a 1 mm sieve, employing an IKA MF10 knife mill. To extract lipophilic compounds, approximately 3–4 g of straw samples were accurately weighed and subjected to Soxhlet extraction with acetone for 8 h. Following extraction, the solvent was carefully evaporated under a vacuum to yield a dry extract that was then accurately weighed. Three replicates were used for each determination.

### Gas Chromatography–Mass Spectrometry (GC–MS)

The lipophilic extracts were redissolved in chloroform for chromatographic analysis. The GC–MS analyses were conducted both underivatized and after derivatization with *N*,*O*-bis(trimethylsilyl)trifluoroacetamide (BSTFA) containing 1% trimethylchlorosilane (TMCS) (Merk, 99% excluding TMCS). The analyses were carried out using a Shimadzu QP 2010 Ultra GC-MS system (Kyoto, Japan) according to the methods published elsewhere.^22^ For compound identification, mass spectra were compared with those published in the Wiley and National Institute of Standards and Technology (NIST) spectral libraries and, whenever feasible, by comparison with authentic standards. Chromatographic peak areas were used to quantify the identified compounds. To determine the concentration of individual compounds, response factors for each compound or closely related compounds were employed. To this end, a calibrated curve was constructed using a mixture of various standards, including tetracosane (Sigma-Aldrich, 99%), palmitic acid (Sigma-Aldrich, 99%), 5α-cholestan-3-one (Sigma-Aldrich, ≥98%), 1-triacontanol (Sigma-Aldrich, ≥98%), cholesta-3,5-diene (Sigma-Aldrich, 95%), sitosterol (Sigma-Aldrich, 99%), cholesteryl linoleate (Sigma-Aldrich, ≥98%), sitosteryl 3β-d-glucopyranoside (Sigma-Aldrich, 75%), 1-monopalmitin (Sigma-Aldrich, ≥99%), 1,3-dipalmitin (Sigma-Aldrich, ≥99%) and tripalmitin (Sigma-Aldrich, ≥99%). Three replicates were analyzed for each sample.

## Results and Discussion

### Composition of Lipophilic Extracts From Oat Straws

**Figure 1:**
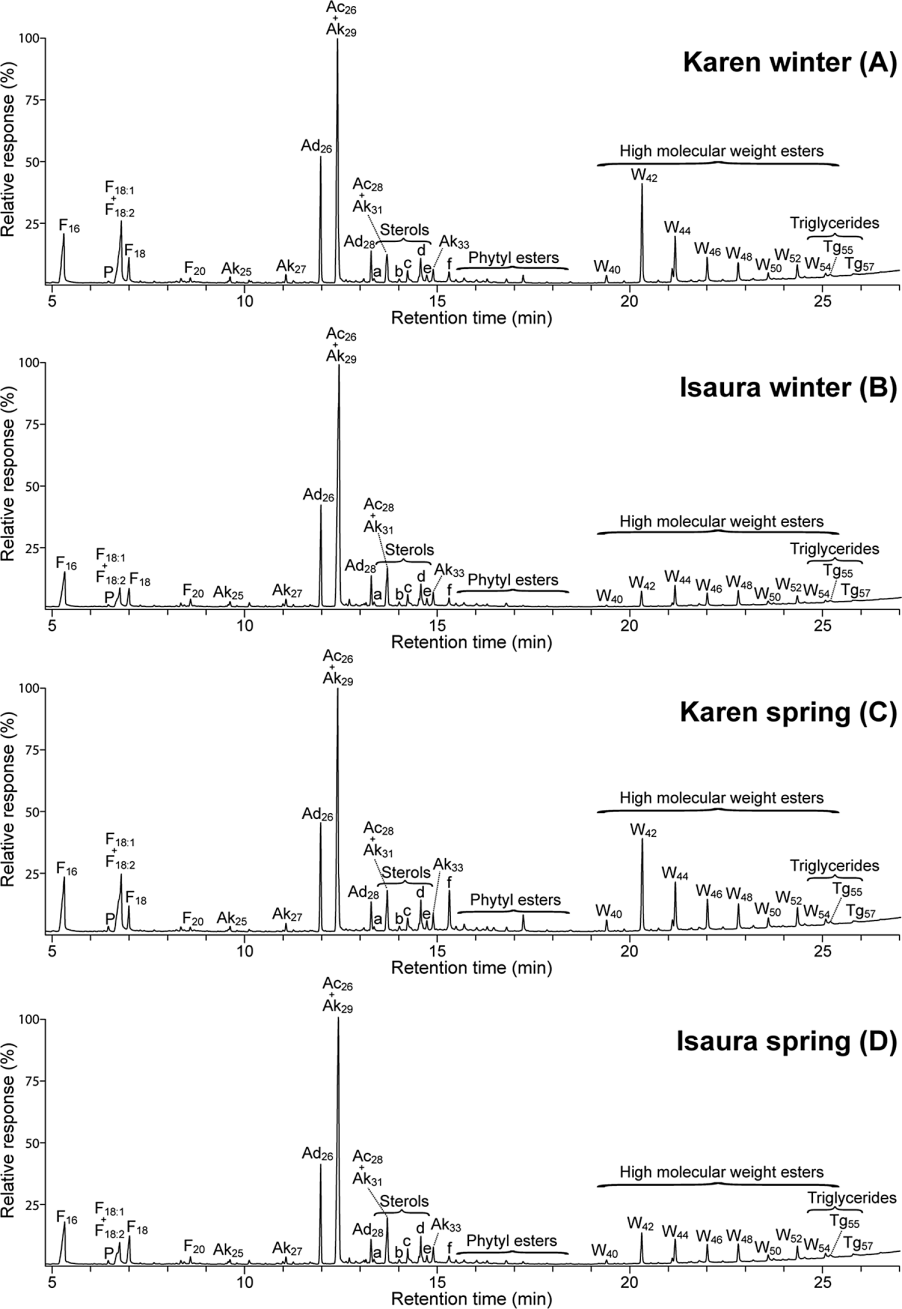
GC-MS chromatograms of the acetone extracts from the straws of Karen and Isaura oat varieties planted in winter (A,B) and spring (C,D). F(n), *n*-fatty acid series; Ak(n), *n*-alkane series; Ad(n), *n*-aldehyde series; Ac(n), *n*-fatty alcohol series; P, phytol; W(n), high molecular weight ester series; and Tg(n), triglyceride series. Labels for selected compounds are a, cholesterol; b, campesterol; c, stigmasterol; d, sitosterol; e, cycloeucalenol; f, 14,16-hentriacontanedione.

**Figure 2:**
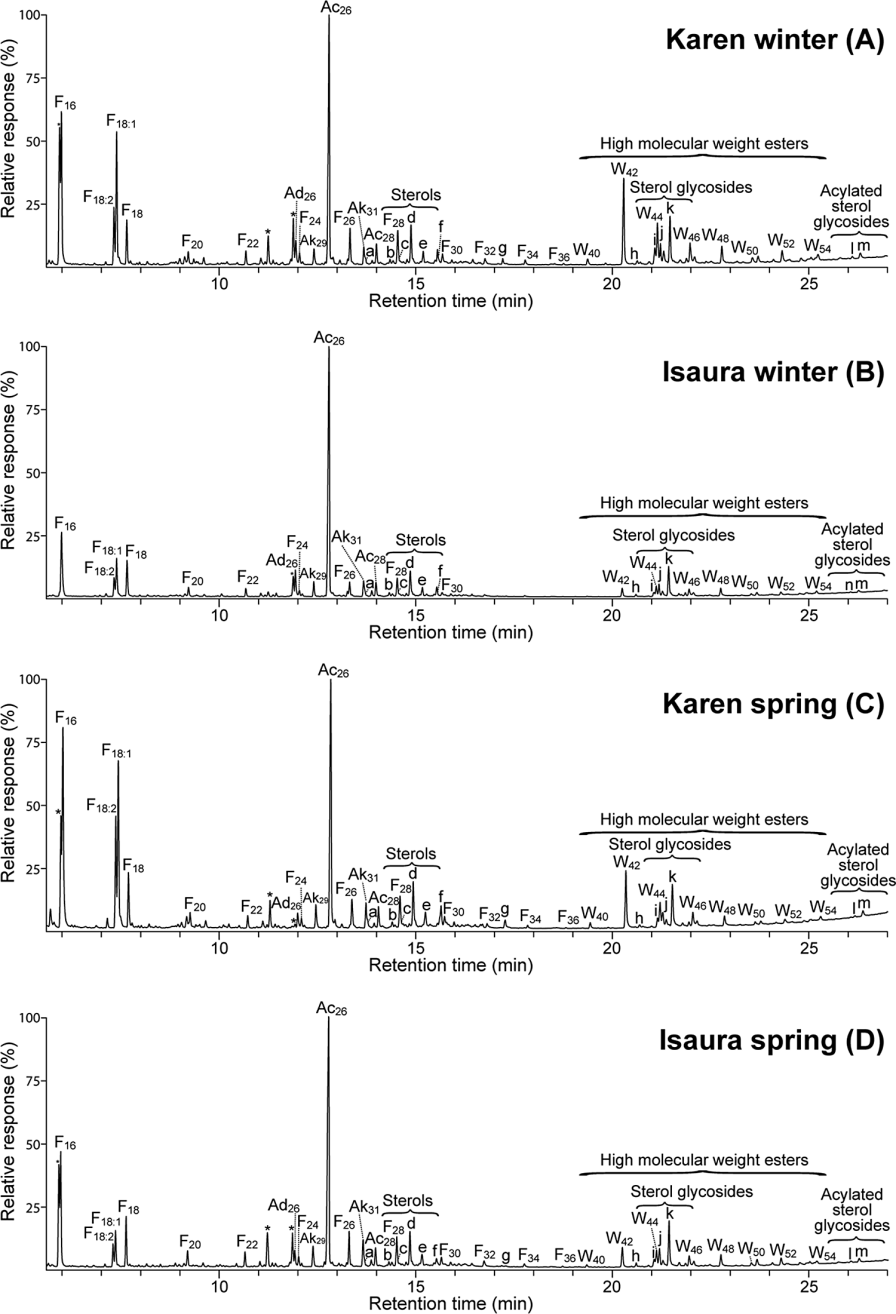
GC-MS chromatograms of the silylated acetone extracts from the straws of Karen and Isaura oat varieties planted in winter (A,B) and spring (C,D). F(n), *n*-fatty acid series; Ak(n), *n*-alkane series; Ad(n), *n*-aldehyde series; Ac(n), *n*-fatty alcohol series; W(n), high molecular weight ester series. Labels for selected compounds are a, cholesterol; b, campesterol; c, stigmasterol; d, sitosterol; e, cycloeucalenol; f, 14,16-hentriacontanedione; g, phytyl linoleate; h, cholesteryl 3β-d-glucopyranoside; i, campesteryl 3-β-d-glucopyranoside; j, stigmasteryl 3β-d-glucopyranoside; k, sitosteryl 3β-d-glucopyranoside; l, stigmasteryl (6′-*O*-palmitoyl)-3β-d-glucopyranoside; m, sitosteryl (6′-*O*-palmitoyl)-3β-d-glucopyranoside.

**Figure 3:**
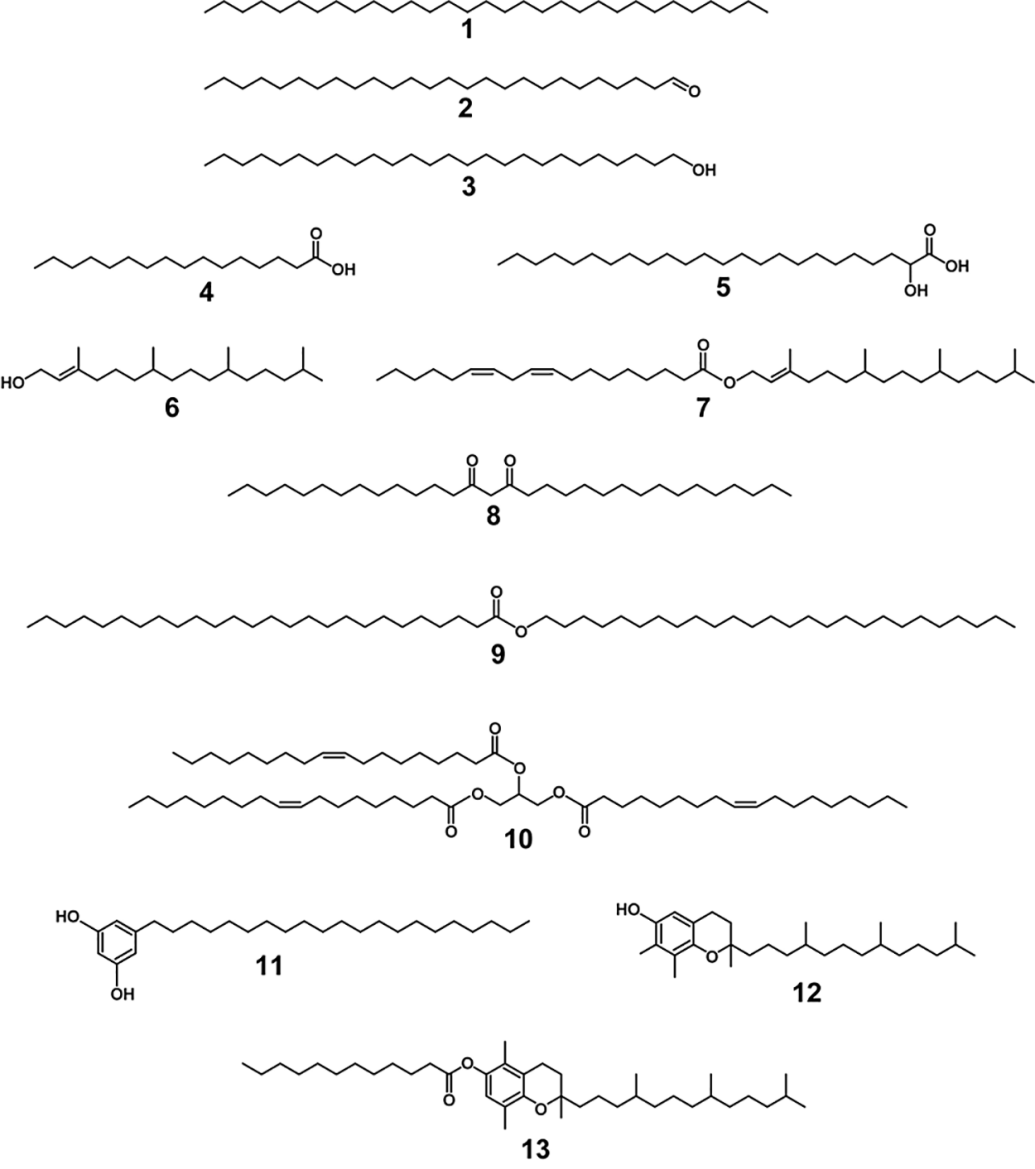
Chemical structures of compounds representative of different families of lipids extracted from the straws of the Karen and Isaura oat varieties. **1**: *n*-hentriacontane; **2**: *n*-hexacosanal; **3**: *n*-hexacosanol; **4**: palmitic acid; **5**: 2-hydroxytetracosanoic acid; **6**: phytol; **7**: phytyl octadeca-9,12-dienoate; **8**: 14,16-hentriacontanedione; **9**: hexacosacanoic acid, hexacosyl ester; **10**: triolein; **11**: 5-*n*-heneicosylresorcinol; **12**: γ-tocopherol; **and 13**: β-tocopheryl dodecanoate.

**Figure 4:**
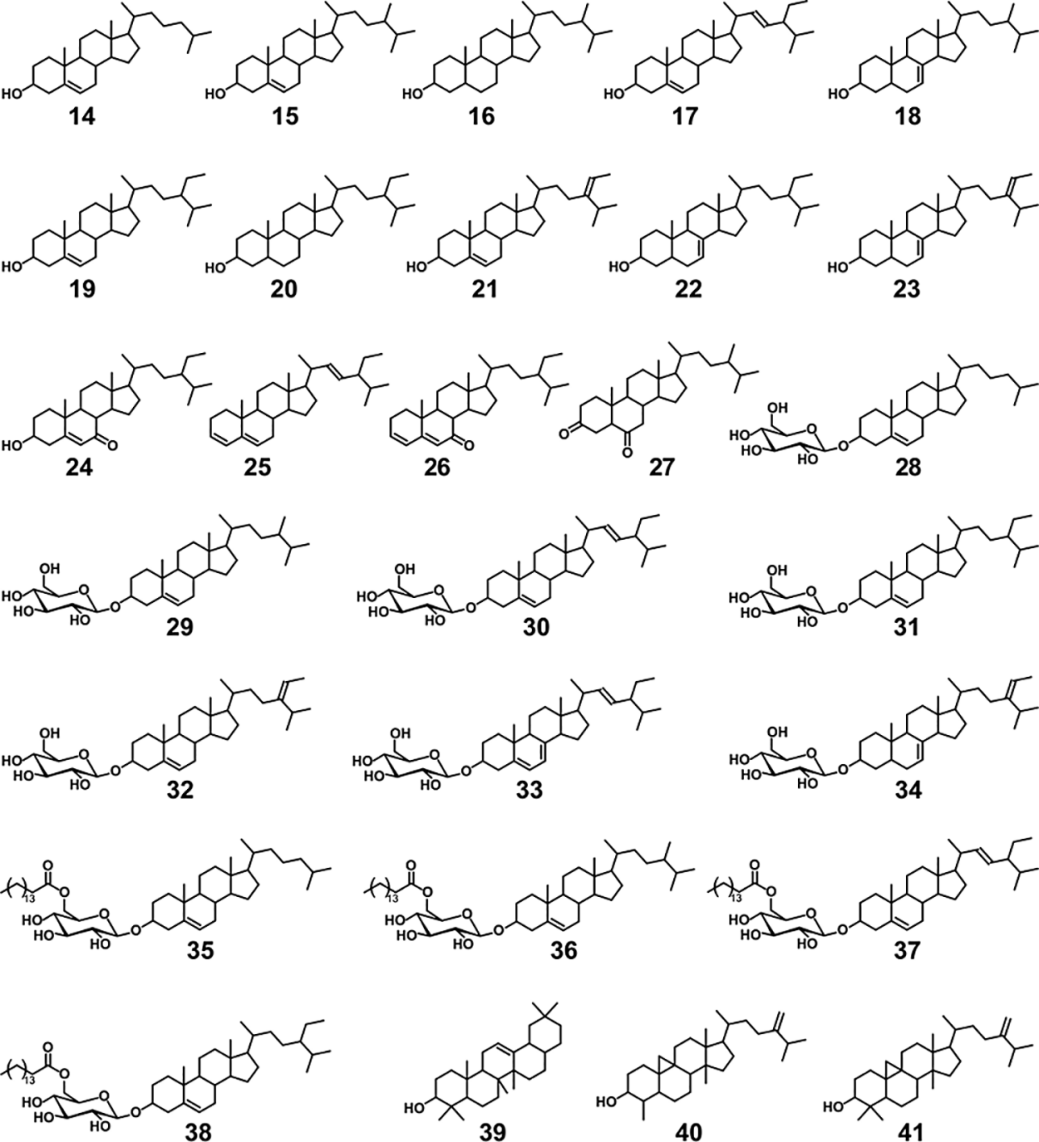
Chemical structures of the main steroid and triterpenoid compounds identified in the acetone extracts of the straws of the Karen and Isaura varieties. Steroid compounds: **14**, cholesterol; **15**, campesterol; **16**, ergostanol; **17**, stigmasterol; **18**, Δ^7^-campesterol; **19**, sitosterol; **20**, stigmastanol; **21**, Δ^5^-avenasterol; **22**, Δ^7^-stigmastenol; **23**, Δ^7^-avenasterol; **24**, 7-oxo-sitosterol; **25**, stigmasta 3,5,22-triene; **26**, stigmasta 3,5-dien-7-one; **27**, stigmastane-3,6-dione; **28**, cholesteryl 3β-d-glucopyranoside; **29**, campesteryl 3β-d-glucopyranoside; **30**, stigmasteryl 3β-d-glucopyranoside; **31**, sitosteryl 3β-d-glucopyranoside; **32**, Δ^5^-avenasteryl 3β-d-glucopyranoside; **33**, Δ^7^-stigmasteryl 3β-d-glucopyranoside; **34**, Δ^7^-avenasteryl 3β-d-glucopyranoside; **35**, cholesteryl (6′-*O*-palmitoyl) 3β-d-glucopyranoside; **36**, campesteryl (6′-*O*-palmitoyl) 3β-d-glucopyranoside; **37**, stigmasteryl (6′-*O*-palmitoyl) 3β-d-glucopyranoside; **38**, sitosteryl (6′-*O*-palmitoyl) 3β-d-glucopyranoside. Triterpenoid compounds: **39**, β-amyrin; **40**, cycloeucalenol; and **41**, 24-methylenecycloartanol.

The contents of lipophilic extractives in the oat straw samples were rather similar, accounting for 2.0% for the Karen variety (for both winter and spring sowing) and 2.1% for the Isaura variety (for both winter and spring sowing). The composition of lipophilic extractives in these oat straws was thoroughly analyzed by GC-MS using medium-length, high-temperature capillary columns, with thin films, according to the method developed by our group that allowed the analysis of a wide range of compounds, from low molecular weight fatty acids to high molecular weight lipids such as sterol esters, sterol glycosides, long-chain esters, and triglycerides.^23,24^ For a complete and more convenient identification of the compounds, the acetone extracts were analyzed both underivatized and as their trimethylsilyl (TMS) ether derivatives. Chromatograms of nonderivatized and silylated straw extracts from both oat varieties are shown in Figures 1 and 2 respectively. The identified compounds encompassed a diverse range, including hydrocarbons, *n*-fatty acids, 2-hydroxy fatty acids, *n*-fatty alcohols, phytol and phytyl esters, high molecular weight esters (waxes), mono-, di-, and triglycerides, steroids (free sterols, ketones, hydrocarbons, glycosides), tocopherols and tocopheryl esters, alkylresorcinols, and β-diketones. The identities and abundances (milligrams per kilogram of dry material) of all compounds identified in the different oat straws are presented in Table 1. Representative structures of the different classes of lipophilic compounds identified are illustrated in Figure 3 (for aliphatics) and Figure 4 (for steroids/triterpenoids).

**Table 1:** Composition and Abundance (Milligrams per Kilogram) of the Lipophilic Compounds Identified in the Acetone Extracts of the Straws of the Karen and Isaura Oat Varieties Planted in Winter and Spring Seasons^a^

compounds	Karen	Karen	Isaura	Isaura
	winter	spring	winter	spring
*n***-alkanes**	**160 ± 31**	**182 ± 18**	**264 ± 22**	**442 ± 8**
*n*-pentacosane	12 ± 4	13 ± 1	23 ± 2	21 ± 1
*n*-heptacosane	16 ± 5	20 ± 2	32 ± 1	33 ± 1
*n*-nonacosane	47 ± 12	55 ± 6	78 ± 4	148 ± 1
*n*-hentriacontane (**1**)	58 ± 3	70 ± 6	93 ± 13	172 ± 0
*n*-tritriacontane	22 ± 5	20 ± 2	33 ± 5	60 ± 4
*n*-pentatriacontane	5 ± 2	4 ± 1	5 ± 1	8 ± 1
*n***-aldehydes**	**1504 ± 74**	**1380 ± 54**	**1940 ± 143**	**1769 ± 50**
*n*-tricosanal	9 ± 2	8 ± 2	11 ± 2	13 ± 2
*n*-tetracosanal	6 ± 2	5 ± 3	12 ± 2	11 ± 1
*n*-pentacosanal	18 ± 6	14 ± 2	26 ± 7	26 ± 1
*n*-hexacosanal (**2**)	1082 ± 32	990 ± 10	1295 ± 53	1146 ± 27
*n*-heptacosanal	8 ± 0	5 ± 1	42 ± 8	53 ± 2
*n*-octacosanal	235 ± 16	209 ± 18	315 ± 44	281 ± 12
*n*-nonacosanal	3 ± 1	3 ± 1	7 ± 1	7 ± 1
*n*-triacontanal	111 ± 8	125 ± 12	200 ± 17	196 ± 1
*n*-dotriacontanal	22 ± 5	14 ± 5	20 ± 7	25 ± 2
*n*-tetratriacontanal	10 ± 2	7 ± 0	12 ± 2	11 ± 1
*n***-fatty alcohols**	**1570 ± 121**	**1267 ± 19**	**2536 ± 99**	**2279 ± 139**
*n*-docosanol	4 ± 0	6 ± 1	4 ± 1	14 ± 3
*n*-tetracosanol	10 ± 2	8 ± 0	21 ± 4	19 ± 1
*n*-pentacosanol	14 ± 3	7 ± 1	19 ± 1	17 ± 1
*n*-hexacosanol (**3**)	1418 ± 106	1177 ± 14	2379 ± 85	2127 ± 133
*n*-heptacosanol	4 ± 0	5 ± 0	8 ± 2	11 ± 0
*n*-octacosanol	120 ± 10	64 ± 3	105 ± 6	91 ± 1
*n***-fatty acids**	**2179 ± 151**	**1861 + 148**	**1339 ± 144**	**1903 ± 97**
*n*-tetradecanoic acid	63 ± 5	86 ± 11	29 ± 5	97 ± 6
*n*-pentadecanoic acid	20 ± 0	20 ± 7	3 ± 0	17 ± 3
*n*-hexadecanoic acid (**4**)	523 ± 45	550 ± 20	484 ± 40	597 ± 19
*n*-heptadecanoic acid	7 ± 1	5 ± 0	7 ± 2	7 ± 1
*cis,cis*-octadeca-9,12-dienoic acid	218 ± 7	232 ± 1	75 ± 7	122 ± 8
*cis-*octadec-9-enoic acid	581 ± 27	454 ± 51	163 ± 15	166 ± 4
*n*-octadecanoic acid	162 ± 12	189 ± 21	148 ± 18	288 ± 30
*n*-nonadecanoic acid	3 ± 1	4 ± 1	5 ± 1	4 ± 1
*n*-eicosanoic acid	54 ± 7	37 ± 7	65 ± 11	73 ± 4
*n*-heneicosanoic acid	4 ± 1	3 ± 1	4 ± 1	7 ± 2
*n*-docosanoic acid	61 ± 2	32 ± 1	59 ± 7	68 ± 2
*n*-tricosanoic acid	21 ± 1	9 ± 2	13 ± 2	16 ± 1
*n*-tetracosanoic acid	45 ± 4	23 ± 0	29 ± 5	49 ± 3
*n*-pentacosanoic acid	10 ± 2	5 ± 1	10 ± 3	23 ± 1
*n*-hexacosanoic acid	143 ± 13	70 ± 12	91 ± 8	116 ± 1
*n*-heptacosanoic acid	2 ± 0	n.d	2 ± 0	3 ± 0
*n*-octacosanoic acid	166 ± 15	95 ± 2	112 ± 11	162 ± 0
*n*-nonacosanoic acid	6 ± 1	3 ± 1	4 ± 1	11 ± 1
*n*-triacontanoic acid	40 ± 3	23 ± 6	19 ± 5	36 ± 2
*n*-dotriacontanoic acid	26 ± 2	12 ± 2	9 ± 0	20 ± 4
*n*-tetratriacontanoic acid	19 ± 1	8 ± 1	7 ± 2	16 ± 3
*n*-hexatriacontanoic acid	5 ± 1	2 ± 0	1 ± 0	5 ± 1
**2-hydroxy fatty acids**	**34 ± 2**	**17 ± 2**	**17 ± 4**	**24 ± 3**
2-hydroxydocosanoic acid	9 ± 1	4 ± 0	4 ± 1	6 ± 0
2-hydroxytetracosanoic acid (**5**)	17 ± 0	10 ± 2	10 ± 2	14 ± 2
2-hydroxyhexacosanoic acid	8 ± 1	3 ± 0	3 ± 1	4 ± 1
**phytol and phytyl esters**	**181 ± 20**	**342 ± 27**	**69 ± 9**	**126 ± 13**
phytol (**6**)	18 ± 0	32 ± 4	15 ± 0	20 ± 2
phytyl hexadecanoate	28 ± 2	49 ± 5	12 ± 3	19 ± 0
phytyl octadeca-9,12-dienoate (**7**)	102 ± 14	204 ± 12	27 ± 5	58 ± 6
phytyl octadec-9-enoate	10 ± 1	23 ± 2	3 ± 0	6 ± 1
phytyl octadecanoate	11 ± 1	15 ± 3	5 ± 0	10 ± 2
phytyl eicosanoate	7 ± 1	12 ± 1	5 ± 1	9 ± 2
phytyl docosanoate	5 ± 1	7 ± 0	2 ± 0	4 ± 0
**β-diketones**	**83 ± 7**	**290 ± 38**	**10 ± 0**	**40 ± 3**
14,16-hentriacontanedione (**8**)	83 ± 7	290 ± 38	10 ± 0	40 ± 3
**high molecular weight esters**	**5371 ± 318**	**3682 ± 326**	**3192 ± 220**	**4022 ± 148**
esters C_40_	163 ± 23	154 ± 2	49 ± 8	111 ± 10
esters C_41_	43 ± 4	37 ± 8	10 ± 1	25 ± 2
esters C_42_ (**9**)	2069 ± 151	1075 ± 79	590 ± 43	905 ± 6
esters C_43_	44 ± 4	45 ± 19	21 ± 3	29 ± 3
esters C_44_	1265 ± 47	759 ± 31	772 ± 51	848 ± 44
esters C_45_	34 ± 3	25 ± 7	24 ± 3	30 ± 3
esters C_46_	530 ± 23	396 ± 24	472 ± 10	515 ± 9
esters C_47_	37 ± 2	30 ± 5	25 ± 6	30 ± 3
esters C_48_	448 ± 22	401 ± 57	568 ± 33	608 ± 32
esters C_49_	37 ± 1	28 ± 4	23 ± 8	34 ± 1
esters C_50_	174 ± 11	177 ± 14	198 ± 18	212 ± 14
esters C_51_	17 ± 2	22 ± 8	14 ± 4	28 ± 2
esters C_52_	391 ± 23	394 ± 64	347 ± 14	461 ± 6
esters C_54_	119 ± 2	139 ± 4	79 ± 18	186 ± 13
**monoglycerides**^b^	**33 ± 6**	**24 ± 5**	**15 ± 2**	**25 ± 4**
1-monopalmitin (1-P)	11 ± 3	10 ± 2	10 ± 1	15 ± 2
1-monolinolein (1-L)	8 ± 1	7 ± 1	2 ± 1	4 ± 0
1-monoolein (1-O)	14 ± 2	7 ± 2	3 ± 0	6 ± 2
**diglycerides**^b^	**113 ± 7**	**49 ± 9**	**n.d**	**7 ± 2**
1,2-Dg37 (1,2-PO + 1,2-PL)	28 ± 2	14 ± 2	n.d	2 ± 0
1,3-Dg37 (1,3-PO + 1,3-PL)	12 ± 1	6 ± 1	n.d	1 ± 0
1,2-Dg39 (1,2-O2 + 1,2-L2 + 1,2-OL)	47 ± 2	19 ± 4	n.d	2 ± 1
1,3-Dg39 (1,3-O2 + 1,3-L2 + 1,3-OL)	26 ± 2	10 ± 2	n.d	2 ± 1
**triglycerides**^b^	**363 ± 39**	**373 ± 36**	**267 ± 24**	**307 ± 13**
Tg53 (P2O + P2S + P2L)	50 ± 11	56 ± 13	38 ± 3	47 ± 9
Tg55 (PL2 + PLS + PO2 + PS2 + PLO + PLS)	137 ± 25	131 ± 21	119 ± 17	101 ± 2
Tg57 (L3 + O3 (**10**))	176 ± 3	186 ± 2	110 ± 4	159 ± 2
*n***-alkylresorcinols**	**13 ± 2**	**15 ± 3**	**5 ± 1**	**8 ± 1**
5-*n*-heptadecylresorcinol	1 ± 0	1 ± 0	tr	tr
5-*n*-nonadecylresorcinol	3 ± 0	3 ± 1	1 ± 0	1 ± 0
5-*n*-heneicosylresorcinol (**11**)	4 ± 1	5 ± 1	2 ± 1	3 ± 1
5-*n*-tricosylresorcinol	3 ± 1	4 ± 1	1 ± 0	2 ± 0
5-*n*-pentacosylresorcinol	1 ± 0	1 ± 0	1 ± 0	1 ± 0
5-*n*-heptacosylresorcinol	1 ± 0	1 ± 0	tr	1 ± 0
**tocopherols and tocopheryl esters**	**26 ± 3**	**86 ± 10**	**36 ± 6**	**71 ± 7**
α-tocopherol (Vit E)	tr	tr	tr	tr
γ-tocopherol (**12**)	4 ± 1	7 ± 1	5 ± 0	5 ± 0
δ-tocopherol	1 ± 0	1 ± 0	1 ± 0	1 ± 0
α-tocopheryl dodecanoate	5 ± 0	10 ± 2	8 ± 2	18 ± 2
α-tocopheryl tetradecanoate	8 ± 1	24 ± 3	11 ± 3	22 ± 1
α-tocopheryl hexadecanoate	1 ± 0	6 ± 1	2 ± 0	3 ± 1
β-tocopheryl dodecanoate (**13**)	1 ± 0	17 ± 1	n.d	2 ± 1
β-tocopheryl tetradecanoate	5 ± 1	17 ± 2	7 ± 1	17 ± 1
β-tocopheryl hexadecanoate	1 ± 0	4 ± 0	2 ± 0	3 ± 1
**sterols**	**504 ± 18**	**457 ± 24**	**675 ± 41**	**672 ± 29**
cholesterol (**14**)	31 ± 2	31 ± 1	43 ± 3	38 ± 1
campesterol (**15**)	28 ± 0	28 ± 2	40 ± 2	37 ± 3
ergostanol (**16**)	14 ± 3	12 ± 0	20 ± 3	17 ± 1
stigmasterol (**17**)	136 ± 1	123 ± 4	166 ± 13	172 ± 10
Δ^7^-campesterol (**18**)	20 ± 0	20 ± 1	30 ± 1	30 ± 4
sitosterol (**19**)	198 ± 3	178 ± 5	272 ± 10	264 ± 8
stigmastanol (**20**)	50 ± 5	48 ± 6	75 ± 5	81 ± 1
Δ^5^-avenasterol (**21**)	2 ± 0	2 ± 0	3 ± 0	2 ± 0
Δ^7^-stigmastenol (**22**)	5 ± 0	4 ± 1	8 ± 0	6 ± 0
Δ^7^-avenasterol (**23**)	2 ± 0	1 ± 1	1 ± 0	1 ± 0
7-oxo-sitosterol (**24**)	18 ± 4	10 ± 3	17 ± 4	24 ± 1
**steroid hydrocarbons**	**13 ± 2**	**13 ± 2**	**7 ± 1**	**12 ± 1**
stigmasta-3,5,22-triene (**25**)	13 ± 2	13 ± 2	7 ± 1	12 ± 1
**steroid ketones**	**21 ± 4**	**11 ± 1**	**14 ± 3**	**17 ± 3**
stigmasta-3,5-dien-7-one (**26**)	3 ± 0	3 ± 1	3 ± 0	4 ± 1
stigmastane-3,6-dione (**27**)	18 ± 4	8 ± 0	11 ± 3	13 ± 2
**sterol glycosides**	**1795 ± 109**	**1442 ± 92**	**1693 ± 104**	**2828 ± 79**
cholesteryl 3β-d-glucopyranoside (**28**)	59 ± 11	39 ± 2	46 ± 3	96 ± 8
campesteryl 3β-d-glucopyranoside (**29**)	224 ± 6	145 ± 4	150 ± 3	274 ± 28
stigmasteryl 3β-d-glucopyranoside (**30**)	526 ± 13	298 ± 8	388 ± 21	720 ± 23
sitosteryl 3β-d-glucopyranoside (**31**)	739 ± 41	650 ± 38	904 ± 57	1370 ± 6
Δ^5^-avenasteryl 3β-d-glucopyranoside (**32**)	13 ± 2	22 ± 3	22 ± 2	48 ± 7
Δ^7^-stigmasteryl 3β-d-glucopyranoside (**33**)	50 ± 13	46 ± 1	51 ± 8	97 ± 1
Δ^7^-avenasteryl 3β-d-glucopyranoside (**34**)	14 ± 4	18 ± 0	26 ± 0	33 ± 1
cholesteryl (6′-*O*-palmitoyl) 3β-d-glucopyranoside (**35**)	12 ± 4	7 ± 1	5 ± 0	9 ± 1
campesteryl (6′-*O*-palmitoyl) 3β-d-glucopyranoside (**36**)	9 ± 3	13 ± 4	7 ± 0	10 ± 1
stigmasteryl (6′-*O*-palmitoyl) 3β-d-glucopyranoside (**37**)	42 ± 9	44 ± 17	30 ± 0	52 ± 2
sitosteryl (6′-*O*-palmitoyl) 3β-d-glucopyranoside (**38**)	107 ± 3	160 ± 13	64 ± 10	119 ± 1
**triterpenoids**	**129 ± 8**	**120 ± 20**	**183 ± 22**	**179 ± 8**
β-amyrin (**39**)	52 ± 5	50 ± 7	85 ± 11	79 ± 1
cycloeucalenol (**40**)	66 ± 2	60 ± 10	87 ± 8	88 ± 4
24-methylenecycloartanol (**41**)	11 ± 1	10 ± 3	11 ± 3	12 ± 3

a Numbers in parentheses refer to the structures depicted in Figure 3 (1–13) and Figure 4 (14–41).

b Labels for mono-, di-, and triglycerides: P, palmitic acid; L, linoleic acid; O, oleic acid; and S, stearic acid.

**Figure 5:**
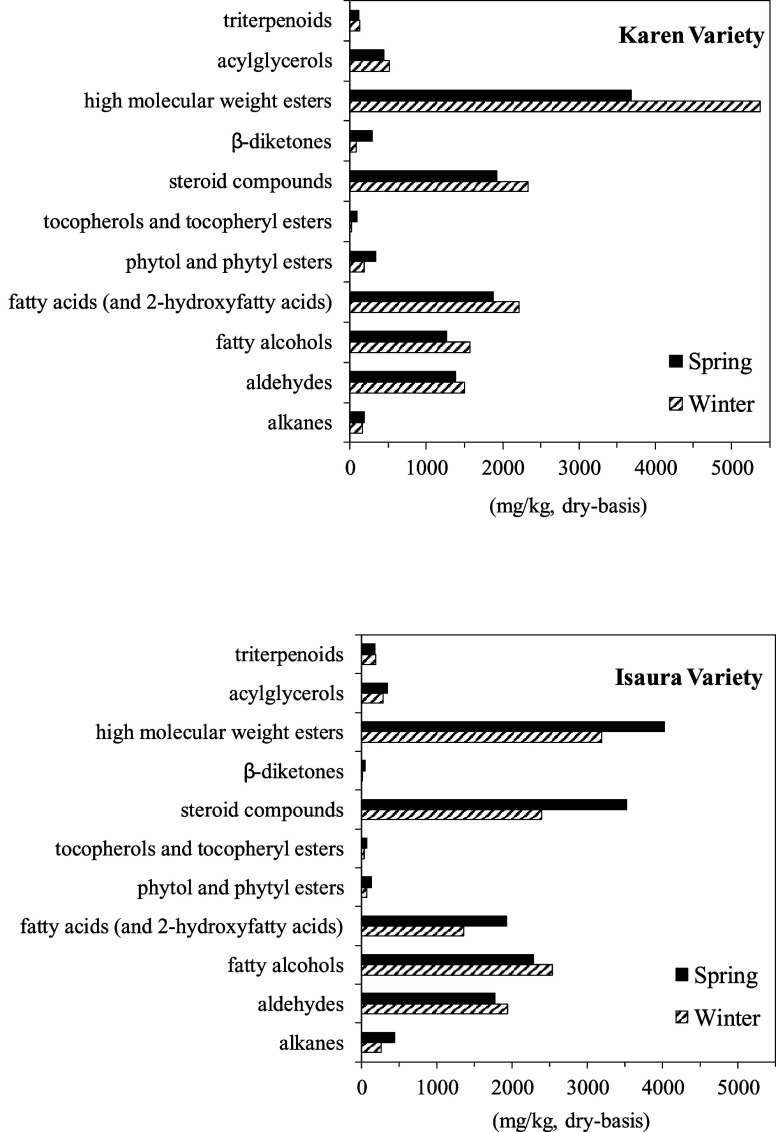
Percentage of the main classes of lipophilic compounds identified in the acetone extracts from straws of Karen and Isaura oat varieties planted in the winter and spring seasons.

The abundances of the different lipid classes in the selected oat straws are indicated in the histograms in Figure 5. Overall, the oat straw extracts were primarily comprised of high molecular weight esters which accounted for up to 3192–5371 mg/kg (26.0–38.1% of all identified compounds), and steroid compounds (1923–3529 mg/kg, 16.6–24.0%), followed by fatty alcohols (1267–2536 mg/kg, 10.9–20.7%), fatty acids (including 2-hydroxyfatty acids; 1356–2213 mg/kg, 11.0–16.2%), and aldehydes (1380–1940 mg/kg, 10.7–15.8%), with lower amounts of acylglycerides (282–509 mg/kg, 2.3–3.8%), alkanes (160–442 mg/kg, 1.1–3.0%), phytol and phytyl esters (69–342 mg/kg, 0.6–2.9%), β-diketones (10–290 mg/kg, 0.1–2.5%), triterpenoids (120–183 mg/kg, 0.9–1.5%), tocopherols and tocopheryl esters (26–86 mg/kg, 0.2–0.7%), and alkylresorcinols (5–15 mg/kg, 0.1%).

The same main families of lipophilic compounds found in oat straw have also been observed in other cereal straws, such as rice and wheat straws,^19,20^ though with notable differences. In rice straw, fatty acids were the most abundant lipophilic compounds (comprising about 41% of the total), while high molecular weight esters only represented 5.8%.^19^ Conversely, in oat straw, high molecular weight esters were the most abundant lipophilic compounds. Wheat straw, on the other hand, contained relatively high amounts of β-diketones (10% of the total lipophilic compounds), particularly 14,16-hentriacontanedione,^20^ compared to only 0.1–2.5% found in the oat straws analyzed here.

### Changes in Lipid Composition According to Oat Variety and Planting Season

The histograms depicted in Figure 5 revealed notable variabilities among the different classes of compounds according to oat variety and sowing season, demonstrating not only the influence of genetic differences but also the influence of environmental factors on the content and composition of oat straw lipids. Basically, when comparing the two oat samples planted in spring with those planted in winter for both Karen and Isaura varieties, there was a significant increase in the content of β-diketones, phytols, tocopherols, and *n*-alkanes, alongside a decrease in the content of *n*-fatty alcohols, and *n*-aldehydes, as clearly observed in the histograms of Figure 5. On the other hand, significant differences were also evident in the lipid composition of the straws according to the oat variety. A different trend was observed in the content of high molecular weight esters, steroid compounds, *n*-aldehydes, *n*-fatty alcohols, and *n*-alkanes, exhibiting higher contents in the Isaura variety planted in the spring compared with the lower amounts observed in the Karen variety planted in the same season. On the other hand, lower contents of acyl glycerols, β-diketones, phytols, and tocopherols were found in the Isaura variety planted in spring compared to the higher contents of these lipophilic compounds detected in the Karen variety planted in the same season. Likewise, winter-planted Isaura and Karen also exhibited differences, with an increase in *n*-alkanes, *n*-aldehydes, and *n*-fatty alcohols in the Isaura variety and a decrease in the content of high molecular weight esters, acylglycerols, β-diketones, phytols, and *n*-fatty acids.

#### Aliphatic Compounds

The series of *n*-alkanes were identified in the range from *n*-pentacosane (C_25_) to *n*-pentatriacontane (C_35_), with *n*-hentriacontane (C_31_; **1**) being the most predominant compound; only the homologues with odd carbon atom numbers were observed (Table 1). The analyses revealed a greater abundance of *n*-alkanes in the Isaura variety than in the Karen variety. Moreover, both oat varieties exhibited increased levels of alkanes when planted in spring (182 mg/kg for Karen, and 442 mg/kg for Isaura) compared to the same varieties planted in winter (160 mg/kg for Karen, and 264 mg/kg for Isaura).

Considerable amounts of *n*-aldehydes were detected in the selected oat straw samples (Table 1). These series were identified in the range from *n*-tricosanal (C_23_) to *n*-tetratriacontanal (C_34_), with a strong predominance of the homologues with even-number carbon atoms, with *n*-hexacosanal (C_26_, **2**) being the most abundant *n*-aldehyde (ranging from 990 to 1295 mg/kg), followed by *n*-octacosanal (C_28_) and *n*-triacontanal (C_30_). The Isaura variety exhibited the highest abundance of *n*-aldehydes, as seen in Table 1. Additionally, their levels increased when planted during the winter season (1504 mg/kg for Karen and 1940 mg/kg for Isaura) compared to those planted in the spring (1380 mg/kg for Karen and 1769 mg/kg for Isaura).

*n*-Fatty alcohols were also found in considerable amounts in the selected oat straws (Table 1). The series were found in the range from *n*-docosanol (C_22_) to *n*-octacosanol (C_28_), with a strong prevalence of the even-number carbon atoms homologues, and with *n*-hexacosanol (**3**) being the most abundant one (ranging from 1177 to 2379 mg/kg). *n*-Fatty alcohols were previously reported as the most abundant family of compounds found in the benzene/chloroform extract in leaf wax of oats.^21^ The Isaura variety exhibited a higher abundance of *n*-fatty alcohols than the Karen variety, and their levels increased when planted in winter (1570 mg/kg for Karen and 2536 mg/kg for Isaura) when compared with the same cultivars planted in spring (1267 mg/kg for Karen and 2279 mg/kg for Isaura).

*n*-Fatty acids were also identified and accounted for 1339–2179 mg/kg (Table 1). The series were found in the range from *n*-tetradecanoic acid (C_14_) to *n*-hexatriacontanoic acid (C_36_), with a strong predominance of the homologues with an even number of carbon atoms. In all cases, the series presented a bimodal distribution, with a maximum for *n*-hexadecanoic acid (C_16_, palmitic acid; **4**), that is the most abundant one (484–597 mg/kg), and a second maximum for *n*-octacosanoic acid (C_28_). Furthermore, significant amounts of the unsaturated *cis,cis*-octadeca-9,12-dienoic (C_18:2_; linoleic acid) and *cis-*octadec-9-enoic (C_18:1_; oleic acid) acids were also detected, with oleic acid being the most predominant (163–581 mg/kg). Studies regarding the distribution of *n*-fatty acids on oat straws remain notably limited compared to the extensive research focused on other oat components like grains and groats.^12,14,25^ Our study revealed a distinct trend in fatty acid content based on the planting season, showing an increase in the Karen variety planted during winter and a corresponding decrease in the Isaura variety during the same season (Figure 5). Minor amounts of 2-hydroxyfatty acids were also found in the oat straws, accounting for 17 to 34 mg/kg (Table 1) and were identified based on its characteristic mass spectra according to previously published studies.^26−28^ The trend observed in the amounts of 2-hydroxytetracosanoic acid (**5**), the most abundant one, in the selected oat straws closely mirrored that of the *n*-fatty acids (Table 1).

The unsaturated isoprenoid alcohol phytol (**6**), along with a series of phytyl esters, were also present in the selected oat straws, accounting for around 69 to 342 mg/kg (Table 1). Their identification was based on the characteristic mass spectra as previously published.^22^ The phytyl esters identified incorporate *n*-fatty acids ranging from C_16_ to C_22_, along with the unsaturated linoleic (C_18:2_) and oleic (C_18:1_) fatty acids, being phytyl octadeca-9,12-dienoate (**7**) the most abundant one. Previous studies have not reported the presence of phytol and phytyl esters in oat samples. However, these compounds have been identified in a variety of plants.^22,29−31^ Phytol and its esters exhibit significant biological activity and are widely used in both the pharmaceutical and cosmetic industries.^32^ Phytol is released during chlorophyll breakdown and, because its toxic properties to membrane, it is channeled either into the synthesis of α- and δ-tocopherol or into esterification with *n*-fatty acids.^31^ As evidenced in Figure 5, the Karen variety exhibited a higher abundance of phytol and phytyl esters in comparison to that of the Isaura variety. Furthermore, the content of these compounds increased when oats are planted in spring, likely due to heightened hydric stress, which could lead to a more pronounced chlorophyll breakdown.

The oat straw samples also contained significant amounts of β-diketone, specifically 14,16-hentriacontanedione (**8**), ranging from 10 to 290 mg/kg (Table 1). The occurrence of 14,16-hentriacontanedione (**8**) was already reported in oat leaves.^21^ This β-diketone was identified by its distinctive mass spectrum, that was identical to that previously published.^20,33^ The Karen variety exhibited the highest abundance of β-diketone, as depicted in Figure 5. Additionally, the data indicate an increase in β-diketone in samples planted in spring. Primary alcohols have been suggested as precursors of β-diketones.^34^ This fact is evident in the two varieties of oat straw studied, where an increase in *n*-fatty alcohols coincides with a decrease in β-diketones (Figure 5). β-diketones are common in the leaves of various grasses and have been recognized as crucial intermediates in the synthesis of important pharmaceutical compounds for treating many pathological disorders, such as cardiovascular diseases, hypertension, obesity and diabetes, among others.^35−38^

High molecular weight esters, commonly referred to as waxes, were the predominant group of lipophilic compounds identified in the acetone extracts of the oat straws, accounting for 3192–5371 mg/kg (Table 1). These esters were found in the range from C_40_ to C_54_ with a strong even-over-odd carbon atoms predominance, and were composed of diverse long-chain *n*-fatty acids esterified to various long-chain *n*-fatty alcohols. Each chromatographic ester peak is constituted of a complex mixture of various long-chain fatty acids esterified to different long-chain fatty alcohols that coelute within the same peak. Identification of the individual esters was accomplished through analysis of their mass spectra as previously reported.^28,39^ The detailed composition and abundance of the individual high molecular weight esters identified in the selected oat straws are shown in Table 2. These esters are made of *n*-fatty acids ranging from C_14_ to C_28_ and *n*-fatty alcohols ranging from C_22_ to C_29_, with a prevalence of C_26_ alcohol (in agreement with the most abundant fatty acids and fatty alcohols detected in the oat straw samples). Among these esters, C_42_ stands out as the most abundant, which is primarily composed of hexacosanoic acid, hexacosyl ester (**9**), present in quantities ranging from 590 to 2069 mg/kg (Table 2). Waxes containing unsaturated fatty acids were also found, with *cis*-octadec-9-enoic acid, hexacosyl ester predominating, in the range from 119 to 304 mg/kg. The identified unsaturated fatty acid was oleic acid (C_18:1_), coinciding with its status as the predominant free unsaturated fatty acid detected in the oat straw samples (Table 1). In plants, high molecular weight esters are generally found on the surface of leaves, fruits, or seeds that protect against water loss, pathogen attack, and ultraviolet light. These compounds hold substantial value as they serve as essential raw materials for producing lubricants, pharmaceuticals, and cosmetics.^40−42^ The Karen variety, particularly when planted in the winter, notably exhibits a high abundance of high molecular weight esters (Table 1). Intriguingly, this aligns with the period when *n*-fatty acids and *n*-fatty alcohols also peak in abundance within the Karen variety. Conversely, in the Isaura variety, waxes are more prevalent during spring, coinciding with an increased abundance in *n*-fatty acids. This period shows minimal disparity in the quantities of *n*-fatty alcohols between spring and winter, although these alcohols are notably abundant in the Isaura variety.

**Table 2:** Composition and Abundance (Milligrams per Kilogram, Dry Basis) of the Different High Molecular Weight Esters Identified in the Acetone Extracts of the Karen and Isaura Oat Straws Planted in Winter and Spring Seasons

compound	fatty acid/fatty alcohol	karen	karen	Isaura	Isaura
		winter	spring	winter	spring
**esters C**_**40**_		**163 ± 23**	**154 ± 2**	**49 ± 8**	**111 ± 10**
tetradecanoic acid, hexacosyl ester	C_14_/C_26_	141 ± 21	140 ± 1	40 ± 6	99 ± 10
hexadecanoic acid, tetracosyl ester	C_16_/C_24_	18 ± 2	11 ± 1	7 ± 2	10 ± 0
octadecanoic acid, docosyl ester	C_18_/C_22_	4 ± 0	3 ± 0	2 ± 0	2 ± 0
**esters C**_**41**_		**43 ± 4**	**37 ± 8**	**10 ± 1**	**25 ± 2**
pentadecanoic acid, hexacosyl ester	C_15_/C_26_	26 ± 3	22 ± 4	6 ± 1	14 ± 1
hexadecanoic acid, pentacosyl ester	C_16_/C_25_	16 ± 1	14 ± 3	3 ± 0	10 ± 1
heptadecanoic acid, tetracosyl ester	C_17_/C_24_	1 ± 0	1 ± 1	1 ± 0	1 ± 0
**esters C**_**42**_		**2069 ± 151**	**1075 ± 379**	**590 ± 43**	**905 ± 6**
hexadecanoic acid, hexacosyl ester (**9**)	C_16_/C_26_	2058 ± 150	1069 ± 378	583 ± 42	897 ± 6
octadecanoic acid, tetracosyl ester	C_18_/C_24_	11 ± 1	6 ± 1	7 ± 1	8 ± 0
**esters C**_**43**_		**44 ± 4**	**45 ± 19**	**21 ± 3**	**29 ± 1**
hexadecanoic acid, heptacosyl ester	C_16_/C_27_	10 ± 0	13 ± 7	4 ± 1	7 ± 1
heptadecanoic acid, hexacosyl ester	C_17_/C_26_	29 ± 2	27 ± 11	12 ± 1	17 ± 2
octadecanoic acid, pentacosyl ester	C_18_/C_25_	5 ± 2	5 ± 1	5 ± 1	5 ± 0
**esters C**_**44**_		**1265 ± 47**	**759 ± 31**	**772 ± 51**	**848 ± 44**
hexadecanoic acid, octacosyl ester	C_16_/C_28_	136 ± 2	102 ± 7	46 ± 8	66 ± 7
octadecanoic acid, hexacosyl ester	C_18_/C_26_	825 ± 25	500 ± 15	607 ± 40	628 ± 31
*cis-*octadec-9-enoic acid, hexacosyl ester	C_18:1_/C_26_	304 ± 20	157 ± 9	119 ± 3	154 ± 6
**esters C**_**45**_		**34 ± 3**	**25 ± 7**	**24 ± 3**	**30 ± 3**
hexadecanoic acid, nonacosyl ester	C_16_/C_29_	7 ± 0	11 ± 3	6 ± 1	7 ± 1
octadecanoic acid, heptacosyl ester	C_18_/C_27_	6 ± 1	4 ± 1	3 ± 1	5 ± 1
nonadecanoic acid, hexacosyl ester	C_19_/C_26_	21 ± 2	10 ± 3	15 ± 1	18 ± 1
**esters C**_**46**_		**530 ± 23**	**396 ± 24**	**472 ± 10**	**515 ± 9**
octadecanoic acid, octacosyl ester	C_18_/C_28_	49 ± 5	38 ± 5	41 ± 0	41 ± 1
eicosanoic acid, hexacosyl ester	C_20_/C_26_	481 ± 18	358 ± 19	431 ± 10	474 ± 8
**esters C**_**47**_		**37 ± 2**	**30 ± 5**	**25 ± 6**	**30 ± 3**
eicosanoic acid, heptacosyl ester	C_20_/C_27_	3 ± 0	2 ± 1	2 ± 1	3 ± 0
heneicosanoic acid, hexacosyl ester	C_21_/C_26_	27 ± 1	19 ± 3	18 ± 5	19 ± 1
docosanoic acid, pentacosyl ester	C_22_/C_25_	3 ± 0	8 ± 1	3 ± 0	5 ± 1
tricosanoic acid, tetracosyl ester	C_23_/C_24_	4 ± 1	1 ± 0	2 ± 0	3 ± 1
**esters C**_**48**_		**448 ± 22**	**401 ± 57**	**568 ± 33**	**608 ± 32**
eicosanoic acid, octacosyl ester	C_20_/C_28_	28 ± 0	29 ± 1	26 ± 0	33 ± 1
docosanoic acid, hexacosyl ester	C_22_/C_26_	412 ± 20	358 ± 56	534 ± 32	569 ± 31
tricosanoic acid, pentacosyl ester	C_23_/C_25_	8 ± 2	14 ± 0	8 ± 1	6 ± 0
**esters C**_**49**_		**37 ± 1**	**28 ± 4**	**23 ± 8**	**34 ± 1**
tricosanoic acid, hexacosyl ester	C_23_/C_26_	37 ± 1	28 ± 4	23 ± 8	34 ± 1
**esters C**_**50**_		**174 ± 11**	**177 ± 14**	**198 ± 18**	**212 ± 14**
docosanoic acid, octacosyl ester	C_22_/C_28_	18 ± 4	20 ± 5	39 ± 4	25 ± 8
tetracosanoic acid, hexacosyl ester	C_24_/C_26_	156 ± 7	157 ± 9	159 ± 14	187 ± 6
**esters C**_**51**_		**17 ± 2**	**22 ± 8**	**14 ± 4**	**28 ± 2**
pentacosanoic acid, hexacosyl ester	C_25_/C_26_	17 ± 2	22 ± 8	14 ± 4	28 ± 2
**esters C**_**52**_		**391 ± 23**	**394 ± 64**	**347 ± 14**	**461 ± 6**
hexacosanoic acid, hexacosyl ester	C_26_/C_26_	391 ± 23	394 ± 64	347 ± 14	461 ± 6
**esters C**_**54**_		**119 ± 2**	**139 ± 4**	**79 ± 18**	**186 ± 13**
hexacosanoic acid, octacosyl ester	C_26_/C_28_	24 ± 2	136 ± 4	17 ± 6	29 ± 0
octacosanoic acid, hexacosyl ester	C_28_/C_26_	95 ± 0	3 ± 0	62 ± 12	157 ± 13

Acylglycerols were also present in the selected oat straws, albeit in substantially lower quantities compared to those reported in oat grains, where triglycerides stand out as the primary lipid fraction.^43−45^ This observation highlights a significant difference in the distribution of lipids between oat straws and grains. Among acylglycerols, triglycerides were prevalent in oat straw, ranging from 267 to 373 mg/kg (Table 1). Triglycerides were found as a complex mixture of various compounds, resulting from the combination of palmitic, linoleic, and oleic acids. Individual triglycerides were distinguished through GC–MS analysis, based on their distinctive mass spectrometric patterns,^46^ and the list of triglycerides identified is shown in Table 1. The prevalent triglycerides identified predominantly comprised oleic and linoleic acids. Among these, Tg57, encompassing triolein (O3, **10**) and trilinolein (L3), emerged as the most abundant, followed by Tg55 (involving palmitoyldilinolein, PL2, and palmitoyldiolein, PO2, among others) and Tg53 (comprising dipalmitoylolein, P2O, dipalmitoylstearin, P2S, and dipalmitoyllinolein, P2L). Diglycerides were detected in smaller amounts, ranging from 0 to 113 mg/kg, and included various compounds resulting from the combination of palmitic, linoleic, and oleic acids occurring in distinct 1,2- and 1,3-positional isomers. Monoglycerides were present in the lowest quantities, spanning from 15 to 33 mg/kg, and included 1-monopalmitin (1-P), 1-monolinolein (1-L), and 1-monoolein (1-O). The quantities of acylglycerols detected in oat straw are significantly lower when compared to other agricultural residues like maize fibers and rice husks, where acylglycerols, along with *n*-fatty acids, emerged as the predominant lipophilic compounds identified in the acetone extracts.^47^ The histograms in Figure 5 indicate a higher abundance of acylglycerides in the Karen variety.

A series of 5-*n*-alkylresorcinols was also identified among the aliphatic lipophilic compounds. The 5-*n*-alkylresorcinols ranged from 5-*n*-heptadecyl (C_17_) to 5-*n*-heptacosylresorcinol (C_27_), with 5-*n*-heneicosylresorcinol (C_21_, **11**) being the most abundant one. 5-*n*-Alkylresorcinols have been identified in the edible portions of various cereals and are commonly reported lipids in wheat bran.^48^ Additionally, they have been detected in brewer’s spent grain.^49^ The analyses revealed that the quantities of *n*-alkylresorcinols are higher in the Isaura variety and rise when oats are sown in the spring. Alkylresorcinols, despite being present in small quantities in the sampled oat straws (5–15 mg/kg), are valued for their noteworthy bioactive properties, particularly in cancer prevention.^50^

Finally, tocopherols and tocopheryl esters were also found in the acetone extracts of the selected oat straws, accounting for a total of 26 to 86 mg/kg (Table 1). Their identification was based on their characteristic mass spectra, as detailed in prior published works.^22,51^ The identified tocopherols included α-, δ-, and γ-tocopherol. Among these, γ-tocopherol (**12**) emerged as the most prominent tocopherol in oat straw samples. However, interestingly, the tocopherol esters ranged from α- and β-tocopheryl dodecanoate (**13**) to α- and β-tocopheryl hexadecanoate, with no apparent presence of γ-tocopheryl esters. Tocopherols are commonly found in various plant-based foods, including vegetable oils and certain cereal grains, such as wheat, barley, and oats. Their biological activity has been extensively documented.^52^ As depicted in the histograms of Figure 5, the quantities of tocopherols and tocopheryl esters rise when oats are planted in the spring. Moreover, no significant variations were observed between the two oat varieties, as illustrated in Figure 5.

#### Steroid Compounds

Significant amounts of steroid compounds were detected in the acetone extracts of oat straw samples, ranging from 1923 to 3529 mg/kg (Table 1). They included free sterols (**14**-**24**), steroid hydrocarbons (**25**), steroid ketones (**26**, **27**), sterol glycosides (**28**–**34**), and acyl sterol glycosides (**35**–**38**) (Figure 4), all recognized as valuable elements within the pharmaceutical and nutraceutical sectors.^53,54^ Among these, sterol glycosides and acyl sterol glycosides emerged as the most prevalent, ranging from 1442 to 2828 mg/kg, followed by free sterols (457–675 mg/kg), and with minor amounts of steroid ketones (11–21 mg/kg) and steroid hydrocarbons (7–13 mg/kg).

Sterol glycosides and acyl sterol glycosides were identified as their TMS-ether derivatives by their mass spectra and by comparison with authentic standards.^55^ The predominant sterol glycoside in the oat straws was sitosteryl 3β-d-glucopyranoside (**31**) (ranging from 650 to 1370 mg/kg), followed by stigmasteryl 3β-d-glucopyranoside (**30**) (298–720 mg/kg) and campesteryl 3β-d-glucopyranoside (**29**) (145–274 mg/kg) (Table 1). Other sterol glycosides present in the oat straws, albeit in minor amounts, were cholesteryl-, Δ^5^-avenasteryl-, Δ^7^-stigmasteryl-, and Δ^7^-avenasteryl 3β-d-glucopyranosides (**32**-**34**). Regarding the acyl sterol glycosides, considerable amounts of sitosteryl (6′-*O*-palmitoyl)-3β-d-glucopyranoside (**38**) were also detected (64–160 mg/kg), with lower amounts of the cholesteryl-, campesteryl-, and stigmasteryl (6′-*O*-palmitoyl)- 3β-d-glucopyranosides. Table 1 shows a clear trend in the amounts of sterol glycosides across the two planting seasons. In the Karen variety, there is an observable increase in sterol glycoside levels when planted in winter, whereas in the Isaura variety, this quantity experiences a decrease.

Among the free sterols, sitosterol (**19**) was the most predominant in both oat varieties, with concentrations ranging from 178 to 198 mg/kg (Karen) and from 264 to 272 mg/kg (Isaura), followed by stigmasterol (**17**), as detailed in Table 1. Additionally, considerable quantities of other free sterols, such as cholesterol (**14**), campesterol (**15**), ergostanol (**16**), Δ^7^-campesterol (**18**), stigmastanol (**20**), and 7-oxo-sitosterol (**24**), were also identified. Only minor amounts of Δ^5^-avenasterol (**21**), Δ^7^-stigmastenol (**22**), and Δ^7^-avenasterol (**23**) were detected in the selected oat straws, consistent with earlier research, which also noted small quantities of avenasterols on oat leaf lipids.^56,57^ The Isaura variety displayed higher sterol levels compared with the Karen variety, although the quantities remained relatively consistent across both planting seasons (Table 1). Minor amounts of steroid ketones and steroid hydrocarbons were also identified. Among the steroid ketones, stigmastane-3,6-dione (**27**) emerged as the predominant compound, with concentrations ranging from 8 to 18 mg/kg (Table 1), together with minor amounts of stigmasta-3,5-dien-7-one (**26**). The sole identified steroid hydrocarbon, stigmasta-3,5,22-triene (**25**), exhibited variations between 7 and 13 mg/kg.

#### Triterpenoid Compounds

Finally, several triterpenoid compounds, namely, β-amyrin (**39**), cycloeucalenol (**40**), and 24-methylenecycloartanol (**41**), were also detected in both oat varieties (ranging from 120 to 183 mg/kg), with the Isaura variety showing slightly higher levels than the Karen variety. Among them, β-amyrin and cycloeucalenol were the most prominent compounds while 24-methylenecycloartanol was present in smaller amounts. No major trends were observed in their content during the planting season.

In conclusion, this study reports a comprehensive chemical analysis of the lipophilic compounds present in oat straw, investigating the variations influenced by genotype and planting season in two different oat varieties cultivated in spring and winter. The predominant lipophilic compounds included high molecular weight esters, steroid compounds, *n*-fatty alcohols, *n*-fatty acids, and aldehydes. Additionally, lower quantities of alkanes, phytol and phytyl esters, acylglycerides, β-diketones, tocopherols and tocopheryl esters, *n*-alkylresorcinols, and 2-hydroxyfatty acids were observed. Notably, these compound classes exhibited variability in their concentrations concerning oat variety and planting season, demonstrating the combined influence of genetic factors and environmental conditions on the composition of the lipophilic compounds in oat straws. Many of the lipophilic compounds identified hold widespread applications across various industries including pharmaceuticals, nutraceuticals, cosmetics, and chemicals. The significant volume of straw generated as waste during oat harvesting emerges as a valuable reservoir of these compounds, offering a strategic resource for utilization in the aforementioned industries. This approach harnesses the potential of compounds derived from oat straw and aligns with a zero-waste philosophy in biomass utilization. Among these compounds, high molecular weight esters hold promise as a sustainable source for biolubricants, while steroid compounds offer notable nutraceutical and health-enhancing properties. Additionally, free fatty acids and acylglycerols offer versatility in producing oils for diverse applications, while steroids, tocopherols, and phytols exhibit important biological activities. In this context, the Karen oat variety is particularly compelling. It yields substantial quantities of these compounds, especially when cultivated in the winter. This period yields heightened levels of high molecular weight esters and *n*-fatty acids, the most prevalent compounds within the acetone extracts obtained from oat straw.
